# Current Evidence on the Antimicrobial Treatment and Chemoprophylaxis of Human Leptospirosis: A Meta-Analysis

**DOI:** 10.3390/pathogens10091125

**Published:** 2021-09-02

**Authors:** Marta Guzmán Pérez, José Javier Blanch Sancho, Juan Carlos Segura Luque, Fernando Mateos Rodriguez, Elisa Martínez Alfaro, Julián Solís García del Pozo

**Affiliations:** 1 Department of Internal Medicine, Albacete University Hospital, 02006 Albacete, Spain; martaguzpe661@gmail.com; 2 Infectious Diseases Unit, Department of Internal Medicine, Albacete University Hospital, 02006 Albacete, Spain; jjblanchs@sescam.jccm.es (J.J.B.S.); jcsegural@sescam.jccm.es (J.C.S.L.); fmateosrodriguez@gmail.com (F.M.R.); elisamartinezalfaro@gmail.com (E.M.A.); 3 Department of Medical Sciences, Albacete School of Medicine, University of Castilla La Mancha, 02008 Albacete, Spain

**Keywords:** Leptospirosis, treatment, antibiotics, chemoprophylaxis, systematic review

## Abstract

Background: Leptospirosis is a worldwide zoonotic infection, and its management needs to be refined. This study aims to discern which antibiotic would be the best option to treat leptospirosis disease and analyze the efficacy of chemoprophylaxis regimens to prevent this illness. Methods: systematic review and meta-analysis on the efficacy of antibiotic treatment and chemoprophylaxis of leptospirosis in humans. Results: Ten clinical trials compared an antibiotic treatment with placebo or other antibiotic treatments in leptospirosis (the most recent one was published in 2007). The meta-analysis shows no effect of penicillin treatment on mortality compared to placebo (OR 1.65; 95% CI 0.76–3.57; *p* = 0.21). There are no differences between penicillin and cephalosporins or doxycycline. Penicillin does not reduce the time of defervescence (MD-0.16; 95% CI (−1.4) –1.08; *p* = 0.80) nor hospital stay (MD 0.15; 95% CI (−0.75)–1.06; *p* = 0.74). Besides, the data did not demonstrate any effectiveness of the use of penicillin in terms of the incidence of oliguria/anuria, the need for dialysis treatment, time to creatinine normalization, incidence of jaundice, or the liver function normalization time. Eight trials have assessed prophylactic treatment against leptospirosis with different strategies. A weekly dose of 200 mg of doxycycline does not show benefit versus placebo regarding the number of new cases of symptomatic leptospirosis (OR 0.20; 95% CI 0.02–1.87; *p* = 0.16). A single dose of doxycycline at exposure to flood water could have a beneficial effect (OR 0.23; 95% CI 0.07–0.77; *p* = 0.02). None of the other chemoprophylaxis regimens tested have shown a statistically significant effect on the number of new symptomatic cases. Conclusion: There is no evidence that antibiotics are a better treatment than placebo regarding mortality, shortening of fever, liver and kidney function, or reduction in the hospital stay. On the other hand, neither doxycycline nor penicillin, nor azithromycin have shown statistically significant differences in preventing symptomatic infection. Well-designed clinical trials, including other antibiotics such as quinolones or aminoglycosides, are urgently needed to improve our understanding of the treatment for this infection, which continues to be a neglected disease.

## 1. Introduction

Leptospirosis is a worldwide zoonotic infection, although its incidence is greater in tropical regions. Leptospira infects more than a million people annually, with approximately 60,000 deaths, and the number of fatal cases is comparable to some other important neglected tropical diseases such as severe dengue or visceral leishmaniasis [1]. In addition, the epidemiology of leptospirosis has been modified by changes in animal husbandry, climate, and human behavior [2]. Individuals living in urban slum environments characterized by inadequate sanitation and poor housing are at high risk of exposure. The global burden of leptospirosis is expected to rise with the increment in urban poor in tropical regions [3].

Most cases have a biphasic clinical presentation: a septicemic phase followed by immune manifestations [4]. In the initial phase, the clinical symptoms are not specific and can mimic a flu-like syndrome, making diagnosis often difficult. Nevertheless, some clinical (muscle pain, cough, conjunctival involvement, and jaundice) and biological features (thrombocytopenia, cholestasis, rhabdomyolysis, and a rise in serum C reactive protein) can help to diagnose leptospirosis and lead to a quick antibiotic therapy before the progression to a severe icterohemorrhagic (Weil’s disease) or respiratory form associated with higher mortality [5].

Management of leptospirosis needs to be refined [6,7]. Delays in diagnosis due to the lack of adequate clinical suspicion, its non-specific symptoms, limited availability of rapid point-of-care diagnostic tests are some of the reasons why its mortality remains significant [7]. There are several diagnostic methods, including direct and serological tests. Serological tests are based on the detection of antibodies against leptospiral antigens. A microscopic agglutination test (MAT) would not be useful in the first stages of the disease when antibodies are not present [8]. Direct diagnostic methods include phase contrast or dark field microscopy, histochemical staining and immunostaining, culture methods, and polymerase chain reaction (PCR) [8].

In regions where leptospirosis is endemic, outbreaks might happen following heavy rainfall (e.g., after cyclones) and floods. The distribution of leptospirosis cases is known to be uneven, and regions of highest incidence have been identified in several contexts [9].

Preventive measures should limit the mammal reservoir and human exposure. Moreover, there is a need to improve the underlying environmental conditions and infrastructure deficiencies in urban slum communities to fight against this neglected disease [10].

Chemoprophylaxis has been considered as a way to protect humans from leptospirosis [9]. Penicillin G sodium (penicillin G) is generally recommended for severe leptospirosis [11]. Nevertheless, other antibiotics such as Doxycycline or Ceftriaxone have been proposed as alternatives to penicillin G [11]. This study aims to discern which antibiotic would be the best option to treat leptospirosis disease and analyze the efficacy of chemoprophylaxis regimens to prevent this illness. We also try to identify existing knowledge gaps on this topic. For this purpose, a systematic review of the treatment and chemoprophylaxis of human leptospirosis has been carried out.

## 2. Material and Methods

### 2.1. Search Strategy

**Figure 1:**
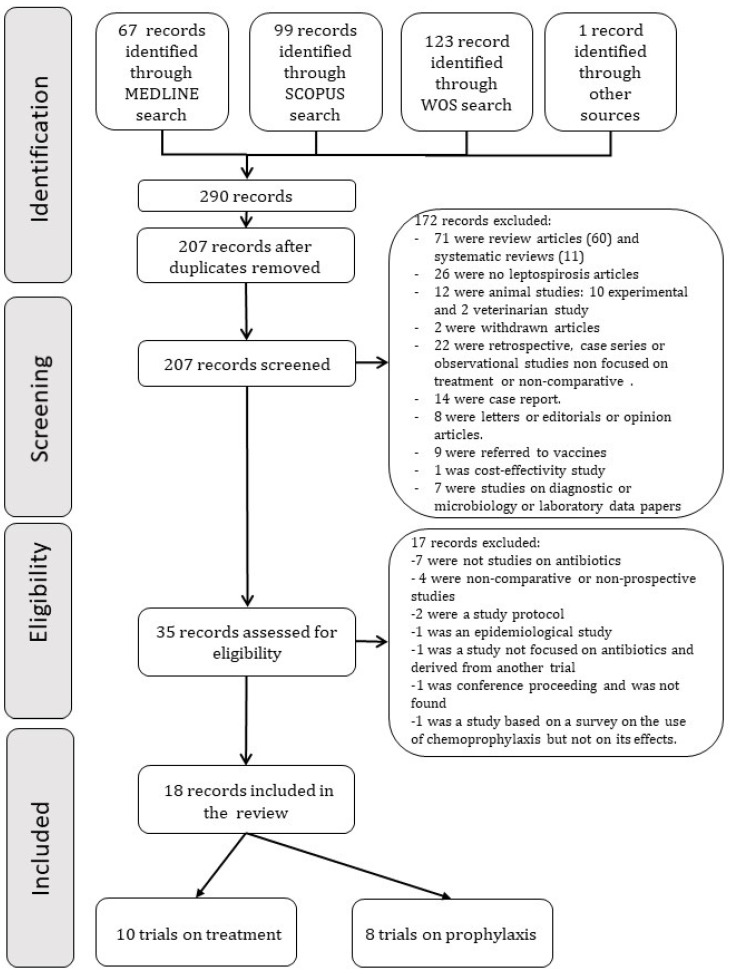
Flow chart of study selection protocol.

A search of all clinical trials comparing any antibiotic for treatment or chemoprophylaxis against human leptospirosis with another antibiotic or placebo until 26 July 2021 was performed. A search was also carried out for trials that looked for the use of antimicrobials for the prophylaxis of *Leptospira* spp. infection in humans. Studies were identified by searching in MEDLINE, Scopus, and Web of Science. The MEDLINE search used the terms “leptospirosis,” “human,” “treatment,” “therapy,” “chemoprophylaxis,” “clinical trial [MesH Terms],” “clinical trial as a topic [MeSH Terms],” “clinical trial,” ”trial.” Scopus and Web of Science search used the same terms. MEDLINE search yielded 67 articles. Search in Scopus and Web of Science yielded 99 and 123 articles, respectively. The selected reports’ bibliographic references were also examined in search of other possible publications not found in the databases mentioned above. When duplicates were removed, 207 articles were screened. Figure 1 shows the search results and the study flow diagram.

### 2.2. Selection Criteria

Clinical trials comparing any antibiotic treatment against human leptospirosis with another antibiotic or placebo or trials looking for the use of any antimicrobial for the prophylaxis of *Leptospira spp*. infection in humans were analyzed. The studies on treatment should meet the following inclusion criteria: (i) well-characterized patients in terms of diagnosis of leptospirosis, (ii) studies should provide data on the treatment administered, duration, and the dose of antimicrobials used and, (iii) well-defined patient outcome. Studies on the prophylaxis of human leptospirosis should meet the following inclusion criteria: (i) the drug administered should be clearly stated as well as the dose and time of administration, (ii) follow-up time should be specified, (iii) it should include the diagnostic criteria for new cases of leptospirosis. Search and selection of studies were performed independently by two investigators (MGP and JSGP).

Those studies without clear diagnostic criteria and methods were excluded. Other exclusion criteria were: studies that did not show in sufficient detail the drug used, the dose, or the duration of treatment. Case reports, case series, non-prospective or non-comparative studies were excluded too, and articles written in languages other than English or Spanish.

### 2.3. Outcome Measures

The parameters considered in the evaluation of the different therapeutic regimens were:

- Duration of hospitalization, defervescence time, kidney failure, liver failure, recovery time from kidney or liver failure, and death;
- For prophylaxis trials, the rate of new cases of leptospirosis in each group was collected.

### 2.4. Data Extraction

Data from studies included in the review were independently extracted by two investigators (MGP and JSGP). Discrepancies were discussed and resolved by consensus. The following variables were collected for each study:

- Author and year of publication;
- Type of study;
- Number of patients;
- Diagnostic criteria;
- Treatment regimens with antibiotics, with its duration, route of administration, and dose;
- If the antibiotic treatment was started as prophylaxis or treatment for the infection;
- Duration of hospitalization, time of defervescence, the incidence of renal or liver failure, recovery time from renal or liver failure, and mortality.

### 2.5. Quality Assessment

**Figure 2:**
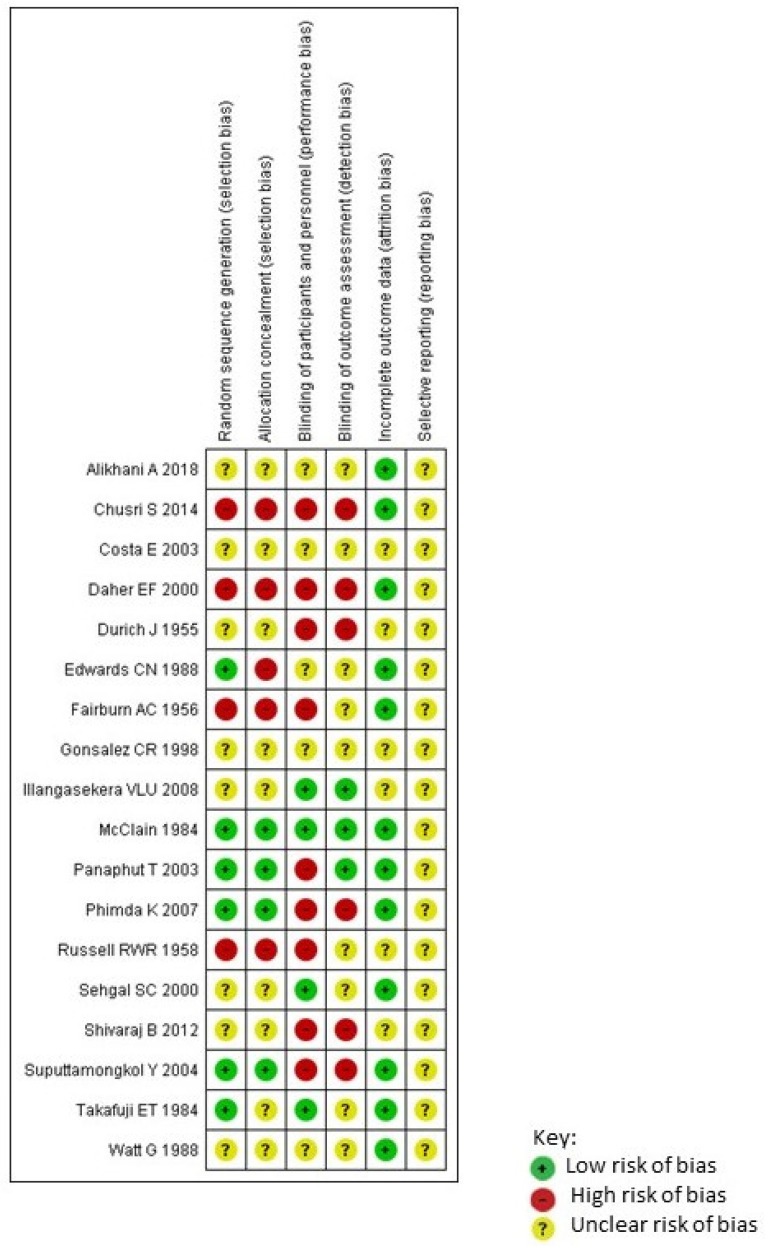
Risk of bias summary: review authors’ judgments about each risk of bias item for each included study.

Randomized clinical trials were assessed by using the Cochrane Risk of Bias Tool for Randomized Controlled Trials [12] independently by two investigators (MGP and JSGP) (Figure 2). Using this method, the risk of different possible biases has been assessed for each included study, and it has been classified as low risk, high risk, or unclear risk of bias [12].

### 2.6. Data Synthesis

The analysis compared the various therapeutic regimens in terms of hospitalization length of stay, time to defervescence, the incidence of renal or liver failure, recovery time from renal or liver failure, and mortality. For prophylaxis trials, the rate of new symptomatic infections was compared among both comparison groups. The differences between the two regimens compared in each case are expressed as an odds ratio with the confidence interval (CI95%) and were contrasted using the Mantel-Haenszel test, using a random effect model which assumes that the effects in the different studies are not identical, treating the differences as random. For continuous variables, the mean difference was used as a measure of effect. Cochran’s Q statistic and the I2 inconsistency statistic were used to measure heterogeneity regarding study results. In all statistical tests, the level of statistical significance used was *p* < 0.05. Analyses were performed using RevMan version 5.4 (The Cochrane Collaboration).

## 3. Results

Ten clinical trials compared an antibiotic treatment with placebo or other antibiotic treatments in leptospirosis [13,14,15,16,17,18,19,20,21,22]. Five of them compared penicillin versus placebo or no antibiotic treatment [13,16,17,18,19]. Other trials compared penicillin with ceftriaxone [20], doxycycline with placebo [15], oxytetracycline with placebo [14], penicillin with cefotaxime, and doxycycline [21], and doxycycline with azithromycin [22]. The most recent trial on treatment was published in 2007 [23]. Among all these trials, they comprised 1071 patients, of whom 396 received penicillin, 168 received a third-generation cephalosporin, 136 received doxycycline, 35 received azithromycin, 31 chloramphenicol, 27 oxytetracycline, and 278 received no antibiotics (178) or received a placebo (100) (Table 1).

**Figure 3:**
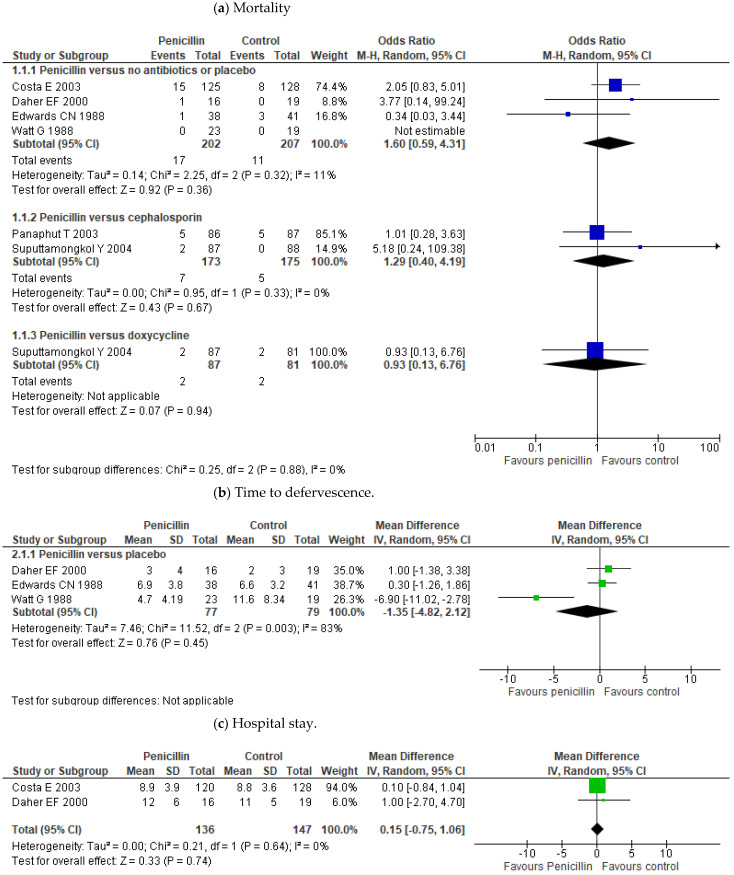
Forest plots on the comparisons on antibiotic treatment efficacy: mortality (**a**), time to defervescence (**b**), and length of hospital stay (**c**). The figure shows the results in treatment and control groups for each study. The sizes of the colored boxes are relative to the study weight. The meta-analysis for each comparison is shown as a diamond.

**Figure 4:**
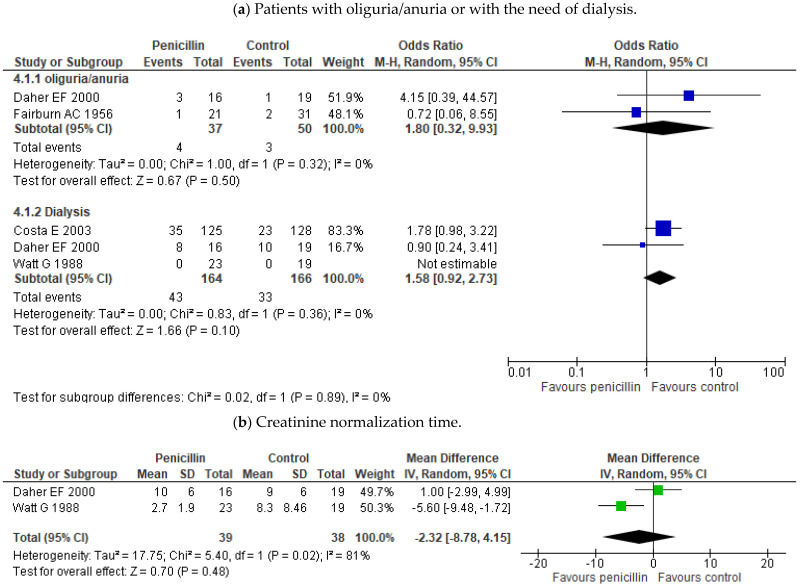
Forest plots of the comparisons on antibiotic treatment effect on renal dysfunction. (**a**) Patients with oliguria/anuria or need of dialysis and (**b**) creatinine normalization time. The figure shows the results in treatment and control groups for each study. The sizes of colored boxes are relative to the study weight. The meta-analysis for each comparison is shown as a diamond.

**Figure 5:**
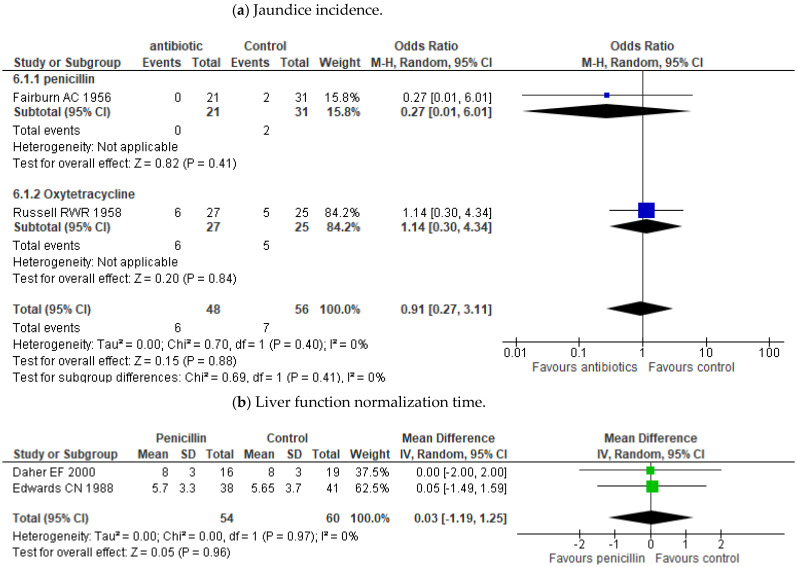
Forest plots of the comparisons on the effect of antibiotics on liver dysfunction. The figure shows the results in treatment and control groups for each study. (**a**) Patients with jaundice (**b**) liver function normalization time.The sizes of colored boxes are relative to the study weight. The meta-analysis for each comparison is shown as a diamond.

Outcome measures of the treatment studies included in this review are in Table 2. The meta-analysis showed no effect of penicillin treatment on mortality compared to placebo or no antibiotic treatment (OR 1.60; 95% CI 0.59–4.31; *p* = 0.36) (Figure 3). Some trials even showed lower mortality in those who received a placebo over penicillin treatment [18,19]. It has not been proven either differences between penicillin and other antibiotic treatments such as cephalosporins (OR 1.29; 95% CI 0.40–4.19; *p* = 0.67) or doxycycline (OR 0.93; 95% CI 0.13–6.76; *p* = 0.94) (Figure 3). Neither effect of penicillin has been demonstrated on the time of defervescence (MD -1.35; 95% CI (−4.82)–2.12; *p* = 0.45) or on hospital stay (MD 0.15; 95% CI (−0.75)–1.06; *p* = 0.74). (Figure 3b,c). Only Watt’s study seemed to show a difference in reducing the duration of fever [16]. Besides, the data also did not demonstrate any effectiveness for the use of penicillin on the incidence of oliguria/anuria, the need for dialysis treatment, or on time to creatinine normalization (Figure 4a,b). There was also no benefit in the incidence of jaundice or in the liver function normalization time (Figure 5a,b).

**Figure 6:**
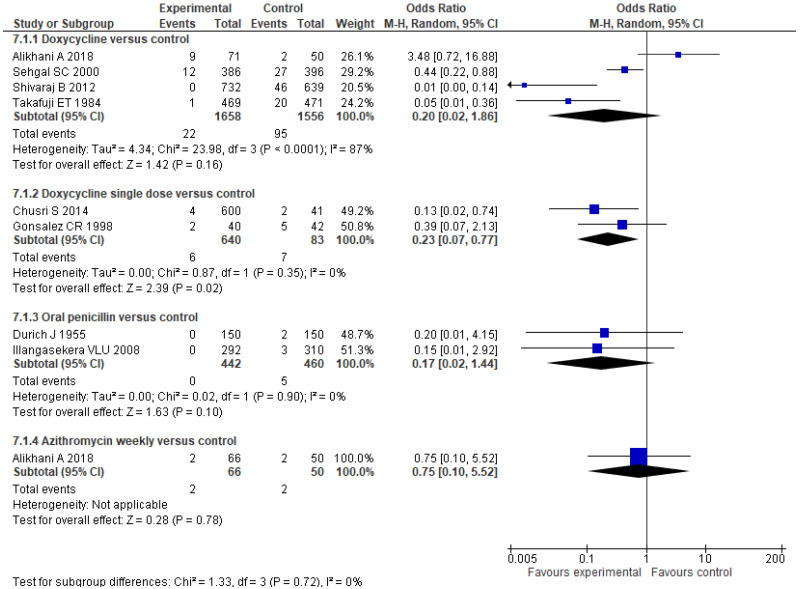
Forest plots of the comparisons on chemoprophylaxis efficacy: number of new symptomatic cases. The figure shows the number of participants and events in treatment and control groups for each study. The sizes of colored boxes are relative to the study weight. The meta-analysis for each comparison is shown as a diamond.

Eight trials assessed prophylactic treatment against leptospirosis with different strategies: four of them with 200 mg of doxycycline weekly [24,26,28,30], two with daily oral penicillin [23,27], another two with a single dose of doxycycline at the time of exposure to flood water [25,29]. One of them also compared doxycycline prophylaxis with a weekly azithromycin regimen [30]. Among all these trials, they comprise 4905 patients. The results of the administration of a weekly dose of 200 mg of doxycycline versus placebo or no antibiotics do not show statistical significance (OR 0.20; 95% CI 0.02–1.87; *p* = 0.16) (Figure 6). There was enormous heterogeneity among the studies. Two of them showed a clear benefit of doxycycline [24,28], but the most recent trial result did not show this benefit [30]. However, this study showed a lower percentage of new IgG seropositivity in those patients who received chemoprophylaxis than in the placebo group (Table 3) [30]. The administration of a single dose of doxycycline at times of exposure reached statistical significance that favors doxycycline (OR 0.23; 95% CI 0.07–0.77; *p* = 0.02) (Figure 6). However, one of the studies included in this latter comparison was not randomized [29]. None of the rest of the comparisons have shown statistical significance in favor of intervention with antibiotics in the prophylaxis of clinical leptospirosis, nor the administration of oral penicillin (OR 0.17; 95% CI 0.02–1.44; *p* = 0.10), nor a regimen that includes azithromycin (OR 0.75; 95% CI 0.10–5.52; *p* = 0.78) (Figure 6), although there seems to be a tendency to have fewer cases of leptospirosis in the groups that have received chemoprophylaxis regimens.

## 4. Discussion

This study provides a meta-analysis on the use of antibiotics in leptospirosis both for treatment and for chemoprophylaxis. The most remarkable thing is that there is a lack of good quality studies on the efficacy of antibiotics at various stages of the disease, and no significant treatment effect has been detected. On the other hand, although most chemoprophylaxis studies show fewer cases of leptospirosis with chemoprophylaxis, they do not reach statistical significance. Therefore, large and good quality studies are needed that consider how leptospirosis was diagnosed and at what stage of the disease treatment is given to detect an effect of treatment.

Another notable fact is the absence of new clinical trials with antibiotics in recent years. The last published trial on treatment was published in 2007 [22], and since that year, no new clinical trials have been published. In addition, several trials were published more than 30 years ago, with a small number of patients and with a non-rigorous methodology regarding the use of placebo or the randomization of patients. A similar result has already been reported by Charan J et al. and Brett-Major DM et al. [31,32]. These authors showed the absence of significant differences between penicillin and placebo in terms of mortality, duration of fever, and renal impairment in leptospirosis. Our work also documents the absence of significant effects in terms of the duration of liver and kidney dysfunction. Besides, our analysis includes information from chemoprophylaxis studies, which gives a complete view of the evidence on the use of antibiotics in this infectious disease. Well-designed studies should be done on the effect of antibiotics at different stages of the disease. Most human diagnostics are serological. MAT has been considered the gold standard for diagnosis [8]. This leads to antibiotics are often started when the immune response is clearing the leptospires. Despite its difficulty, this data justifies studies to describe the effect of antibiotics at different times: exposure, leptospiraemic, and immune phases. The paucity of recent, well-designed trials confirms the idea that leptospirosis remains a neglected tropical disease.

In recent years, trials have been carried out to verify the effect of non-antibiotic treatments such as corticosteroids [33,34] or other drugs [35] in severe leptospirosis, although without a clear result at the moment. On the other hand, although other authors have advocated testing other antibiotics such as quinolones [36], no trials have been conducted for this purpose.

Regarding prophylaxis, the results did not show statistical significance in most comparisons. The antibiotics tested for prophylaxis are the same as those tested for treatment, although some prophylaxis trials being more recent than those ones carried out for treatment. On the other hand, the design of the trials is very uneven in the time of prophylaxis (single or weekly dose). Only when a single dose of doxycycline was used at the moment of floodwater exposition, chemoprophylaxis showed benefit with statistical significance. However, this effect is based on the results of a non-randomized study [29]. However, in most studies, there seems to be a tendency to have fewer cases of leptospirosis in the groups with chemoprophylaxis regimens. Only the Alikhani study [30] shows more cases of clinical leptospirosis in the doxycycline chemoprophylaxis group than in the control group. Nevertheless, in this study, there are fewer cases of new IgG seropositivity patients in the doxycycline group. Probably large studies are needed to clarify the role and efficacy of chemoprophylaxis in human leptospirosis.

Many unanswered questions remain regarding the treatment and prophylaxis of leptospirosis. It has been suggested that early treatment could be more effective, but it is not known from what day in the clinical course (if there is one) the treatment becomes less effective. It is also not known whether other antibiotics such as quinolones or aminoglycosides may play a role. Only randomized trials with a sufficient number of patients can answer these questions. Meanwhile, in the absence of better evidence, treatment with penicillin and doxycycline continues to be recommended in medical texts, but there is no substantial progress on this issue in the last 30 years. This fact confirms that leptospirosis continues to be a neglected disease.

## Figures and Tables

**Table 1:** Studies included in the present review.

Author (Year) and Reference	Treatment/Prophylaxis	Type of Study	Patients	Treatment Groups	*n*	Outcomes
Fairburn AC (1956) [13]	Treatment	Controlled trial	Soldiers admitted to one of two military hospitals in Malaya with agglutination test positive.	Penicillin 600,000 UI/6h versus chloramphenicol 0.5 g/6 h versus no antibiotics (for at least five days)	21 vs. 31 vs. 31	Duration of fever Duration of symptoms “After-peaks” of pyrexia Complications: Jaundice, oliguria, anuria, and uremia
Ross Russell RW (1958) [14]	Treatment	Placebo-controlled trial	52 consecutive patients admitted to the military hospital with leptospirosis confirmed by blood-culture or serological tests.	Oxytetracycline 1.5 g followed by 0.5 g/6 h for at least 5 days versus placebo	27 vs. 25	Duration of pyrexia; Duration of symptoms; Incidence of jaundice; Urea levels.
McClain (1984) [15]	Treatment	Randomized, double-blind controlled trial	Any febrile patient returning from jungle training school without another cause of fever identified and isolation of leptospira in blood or urine, or a fourfold rise in serum antibody titers.	Doxycycline 100 mg/12 h for 7 days versus placebo	14 vs. 15	Duration of fever; Duration of signs and symptoms; Negativization of Leptospira in cultures and MAT.
Watt G (1988) [16]	Treatment	Double-blind placebo-controlled trial	Patients (>16 years) with a high likelihood of leptospirosis and also have leptospirosis confirmed with a fourfold or greater rise in antibody titer by microagglutination test or isolation in blood or urine.	Penicillin G 1.5 MU/6h for 7 days versus placebo	23 vs. 19	Duration of fever; Duration of symptoms. Duration of hospital stay; Number of patients from whom leptospires were isolated after treatment; Raised serum creatinine levels, hepatic tenderness, and hematological and biochemical variables.
Edwards CN (1988) [17]	Treatment	Randomized controlled trial	Patients with a history and physical findings compatible with symptoms of leptospirosis and have a fourfold rise in titer or an initial titer ≥600 in the MAT; a titer 1:80 IgM on an ELISA; a 4+ Patoc I antigen titer; positive leptospira cultures.	Penicillin 2 MU/6 h iv 5 days vs. placebo	38 vs. 41	Time to defervescence; Time to return of biochemical normality; Absence or positive urine culture; Complications; Mortality; Iritis.
Daher EF (2000) [18]	Treatment	Probably non randomized ^†^	Patients with leptospirosis and acute renal failure (plasma creatinine > 1.5 mg/dL) and jaundice on admission. Diagnosis of leptospirosis clinical ald IgM ≥ 1:400 on the 15th day after the onset of symptoms.	Penicillin 6 MU/day iv for 8 days vs. no antibiotics	16 vs. 19	Days of hospitalization; Duration of fever Time to normalization of creatinine, bilirubin (or one-third of maximum value), and platelet count; Dialytic treatment; Values of serum and urine biochemical parameters; Hospital mortality; Presence of oliguria; Positive fluid balance (%); Episodes of bleeding; X-ray evidence of pneumonitis.
Costa E (2003) [19]	Treatment	Randomized controlled trial	Patients with> 4 days with symptoms; ≥26 points in the WHO probability score for leptospirosis; Macroscopic slide test, microagglutination test, and blood culture for microbiologic confirmation in Salvador.	Penicillin 6 mU/day (1 mU/4 h) for seven days versus control (no antibiotic)	125 vs. 128	In-Hospital death; Length of hospital stay.
Panaphut T (2003) [20]	Treatment	Randomized trial. Open-label	Patients (>16 years) with severe leptospirosis, defined by the presence of jaundice, a serum creatine level of >180 µmol/L, or a mean arterial pressure <70 mmHg. Serology diagnostic. WHO criteria.	Ceftriaxone 1 g/day for 7 days versus penicillin G 1.5 MU/6 h for 7 days *.	87 vs. 86	Time to fever resolution Hospital mortality Time to resolution of organ dysfunction (renal failure, hepatic dysfunction, and thrombocytopenia)
Suputtamongkol Y (2004) [21]	Treatment	Randomized, open trial	Patients with suspected severe leptospirosis in some hospitals in Thailand, whom had isolated leptospires from blood or a 4′fold or greater increase in the antibody titer or a single or stable antibody titer of ≥1:400.	Penicillin G 1.5 MU/6 h versus Cefotaxime 1 g/6 h versus Doxycycline 200 mg first dose followed by 100 mg/12 h iv. 7 days **.	181 vs. 172 vs. 187 (87 vs. 81 vs. 88 with confirmed leptospirosis)	Mortality; Time to defervescence; Duration of hospitalization.
Phimda K (2007) [22]	Treatment	Randomized trial	Patients with suspected Leptospirosis and scrub typhus in Thailand Leptospirosis: isolation from blood or positive serologic test (fourfold or greater rise in antibody titer or at least 1:400 on a single specimen).	Doxycycline 200 mg the first dose followed by 100 mg/12 horas seven days versus Azithromycin 1 g initially followed by 500 mg once daily for 2 days.	34:35 (only leptospirosis)	Time of fever clearance; % of afebrile patients at 48 h post-treatment.
Durich J (1956) [23]	Chemoprophylaxis	Probably non randomized ^†^	Rice reapers in the province of Valencia (Spain)	Penicillin procain 100,000 U/12 h vo (for 100 reapers) or dipenicillin N,N’dibenciletilendiamine 100000U/12 h (for 50 reapers) versus control.	150 vs. 150	New cases of leptospirosis.
Takafuji ET (1984) [24]	Chemoprophylaxis	Double-blind, placebo-controlled trial	Healthy volunteers at Jungle Operations Training Center (Panama).	Doxycycline 200 mg weekly vs. placebo.	469:471	New cases of leptospirosis.
Gonsalez CR (1998) [25]	Chemoprophylaxis	Double-blind, randomized, placebo-controlled trial	Residents of a small community in Sao Paulo region, an area at high risk for flooding.	Doxycycline 200 mg single dose vs. placebo.	40 vs. 42	New confirmed cases (symptomatic). Seropositivity
Sehgal SC (2000) [26]	Chemoprophylaxis	Randomized controlled-trial	Healthy persons (>10 years old) residing in Diglipur (North Andaman), India.	Doxycycline 200/week versus placebo (12 weeks).	386 vs. 396	New cases of leptospirosis. Seropositivity
Illangasekera VLU (2008) [27]	Chemoprophylaxis	Randomized, double-blind Placebo-controlled trial	Healthy persons (male farmers) between 20–80 years of age from Central Province, Sri Lanka.	Oral Penicillin 500 mg/bid versus placebo during 1 month.	292 vs. 310	New cases of leptospirosis.
Shivaraj B (2012) [28]	Chemoprophylaxis	Randomized controlled trial	Paddy field farmers in Karnataka state, India.	Doxycycline 200 mg/week for 5 weeks and Information Education Communication activity versus none treatment.	732 vs. 639	New cases of leptospirosis.
Chusri S (2014) [29]	Chemoprophylaxis	Non-randomized trial	Residents in Hat Yai City (Thailand), aged 18 years or above and exposed to flood water.	Doxycycline 200 mg single dose versus no treatment.	600 vs. 41	Seroconversion New cases of symptomatic leptospirosis.
Alikhani A (2018) [30]	Chemoprophylaxis	Randomized, double-blind, placebo-controlled trial	Paddy field workers (from 18 to 65 years); Three endemic cities for leptospirosis in Mazandaran in the north of Iran.	Azithromycin 500 mg/week versus doxycycline 200 mg/week versus placebo during 1 month.	66 vs. 71 vs. 50	Cases of leptospirosis (symptomatic); Seropositivity for leptospirosis.

* Gentamicin was also administered for patients in the penicillin group for whom septicemia as Gram-negative pathogens could not be initially excluded. ** Parenteral study treatment was continued until the patient was afebrile and was well enough to have treatment switched to oral therapy. Treatment was then switched to either oral amoxicillin 2 g/day (for penicillin and cefotaxime groups) or oral doxycycline 200 mg/day (for doxycycline group). ^†^ It is not clear whether these two studies were randomized or not. They are probably non-randomized.WHO: World Health Organization; MAT: microagglutination test; ELISA: Enzyme-Linked ImmunoSorbent Assay.

**Table 2:** Outcome measures of the treatment studies included in this review.

Author (Year) and Reference	Treatment Groups	Mortality	Duration of Fever	Hospital Stay	Oliguria/Anuria	Dialysis	Creatinine Normalization Time	Jaundice	Liver Function Tests Normalization Time
Fairburn AC (1956) [13]	Penicillin 600,000 U/6 h	NR	7.6	NR	1 (4.8%)	NR	NR	0 (0%)	NR
	Chloramphenicol 0.5g/6h	NR	8.8	NR	1 (3.2%)	NR	NR	1 (3.2%)	NR
	control	NR	9	NR	2 (6.5%)	NR	NR	2 (6.5%)	NR
Ross Russell (1958) [14]	Oxytetracycline 1.5g followed by 0.5g/6h for at least 5 days	NR	6.4	NR	NR	NR	NR	6 (22.2%)	NR
	placebo	NR	9.4	NR	NR	NR	NR	5 (20%)	NR
McClain (1984) [15]	Doxiciclina 100 mg cada 12 horas durante 7 días	NR	3.7 ± 0.3	NR	NR	NR	NR	NR	NR
	placebo	NR	5.4 ± 0.3	NR	NR	NR	NR	NR	NR
Watt G (1988) [16]	Penicillin G 1.5 MU/6h for 7 days	0/23	4.7 ± 4.19	NR	NR	0 (0%)	2.7±1.9	NR	NR
	placebo	0/19	11.6 ± 8.34	NR	NR	0 (0%)	8.3±8.46	NR	NR
Edwards CN (1988) [17]	Penicillin 2 MU/6h iv 5 days	1/38	6.9 ± 3.8	NR	NR	NR	NR	NR	5.7 ± 3
	placebo	3/41	6.6 ± 3.2	NR	NR	NR	NR	NR	5.65 ± 3.7
Daher EF (2000) [18]	Penicillin 6 MU/day iv for 8 days	1/16	3 ± 4	12 ± 6	3 (19%)	8 (50%)	10±6	NR	8 ± 3
	no antibiotics	0/19	2 ± 3	11 ± 5	1 (5%)	10 (52%)	9±6	NR	8 ± 3
Costa E (2003) [19]	Penicillin 6 mU/day (1 mU/4h) for seven days	15/125	NR	8.9 ± 3.9	NR	35 (28%)	NR	NR	NR
	control	8/128	NR	8.8 ± 3.6	NR	23 (18%)	NR	NR	NR
Panaphut T (2003) [20]	penicillin G 1.5 MU/6h for 7 days	5/86	3 *	NR	NR	NR	NR	NR	NR
	Ceftriaxone 1 g/day for 7 days	5/87	3 *	NR	NR	NR	NR	NR	NR
Suputtamongkol Y (2004) [21]	Penicillin G 1.5 MU/6h 7 days	2/87	72 (12–240)	6 (2–21)	NR	NR	NR	NR	NR
	Cefotaxime 1 g/6 h 7 days	0/88	60 (8–192)	5.5 (3–37)	NR	NR	NR	NR	NR
	Doxycycline 200 mg first dose followed by 100 mg/12 h iv. 7 days.	2/81	72 (12–264)	5 (2–28)	NR	NR	NR	NR	NR
Phimda K (2007) [22]	Doxycycline 200 mg the first dose followed by 100 mg/12 horas seven days	NR	45 h (8–118 h)	NR	NR	NR	NR	NR	NR
	Azithromycin 1g initially followed by 500 mg once daily for 2 days.	NR	40 h (8–136 h)	NR	NR	NR	NR	NR	NR

Data are expressed as *n* (%) for categorical variables and mean ± standard deviation or median (range) for continuous variables. All continuous variables are expressed in days except when it is indicated in hours (h). * Median. NR: not reported.

**Table 3:** Outcome measures of the chemoprophylaxis studies included in this review.

Author (Year)	Treatment Groups	New Symptomatic Cases	Seroconversion
Durich J (1956) [23]	Penicillin procain 100,000 U/12 h vo or dipenicillin N,N’dibenciletilendiamine	0/150 (0%)	NR
	Control	2/150 (1.3%)	NR
Takafuji ET (1984) [24]	Doxycycline 200 mg semanales (3 weeks aprox)	1/469 (0.2%)	NR
	placebo	20/471 (4.2%)	NR
Gonsalez CR (1998) [25]	Doxycycline 200 mg single dose	2/40 (5%)	IgM: 13/40 (32.5%)
	placebo	5/42 (11.9%)	IgM: 11/42 (26.2%)
Sehgal SC (2000) [26]	Doxycycline 200/week (12 weeks)	12/386 (3.1%)	112/386 (29%)
	placebo	27/396 (6.82%)	101/396 (25.5%)
Illangasekera VLU (2008) [27]	Oral Penicillin 500 mg/bid for one month	0/292 (0%)	NR
	placebo	3/310 (1%)	NR
Shivaraj B (2012) [28]	Doxycycline 200 mg/week for 5 weeks and IEC	0/732 (0%)	NR
	No treatment	46 */639 (7.29%)	NR
Chusri S (2014) [29]	Doxycycline 200 mg single dose	4/600 (0.7%)	17/600 (2.8%)
	No treatment	2/41 (4.9%)	5/41 (12.2%)
Alikhani A (2018) [30]	Azithromycin 500 mg/week	2/66 (3%)	IgM: 2/66(3%) IgG: 5/66 (7.6%)
	doxycycline 200 mg/week	9/71 (12.6%)	IgM: 9/71 (12.6%) IgG: 8/71 (11.3%)
	placebo	2/50 (4%)	IgM: 2/50 (4%) IgG: 12/50 (24%)

Data are expressed as the number of new symptomatic cases/total patients included (%), or the number of new positive antibody cases/total patients included (%). NR: not reported. * This study gives percentage and no the number of new symptomatic cases. 46 was deduced from the data of this study.
